# Dose-Response Effect of Isometric Force Production on the Perception of Pain

**DOI:** 10.1371/journal.pone.0088105

**Published:** 2014-02-04

**Authors:** Gaurav Misra, Tiffany A. Paris, Derek B. Archer, Stephen A. Coombes

**Affiliations:** Laboratory for Rehabilitation Neuroscience, Department of Applied Physiology and Kinesiology, University of Florida, Gainesville, Florida, United States of America; University of Ottawa, Canada

## Abstract

Isometric contractions can influence the way that we perceive pain, but conclusions on the dose-response effect of force amplitude on pain perception are limited because previous studies have not held the duration of force contractions constant while varying force amplitude. To address this issue we designed an experiment that allowed us to accurately guide the amplitude of an isometric pinch grip force contraction on a trial-by-trial basis, while a thermal pain eliciting stimulus was simultaneously delivered for the same duration to the non-contracting hand. Our results show that an increase in the amplitude of force produced by one hand corresponded with a decrease in pain perception in the opposite hand. Our observations provide novel evidence that the centralized inhibitory response that underlies analgesia is sensitive to and enhanced by stronger isometric contractions.

## Introduction

There is a long history of studying the effects of exercise on pain perception (for review see [1], [2], [3]). Exercise can reduce the perception of pain-eliciting mechanical, thermal, and electrical stimuli [4], [5], [6], [7], [8], [9], [10]. Although the most studied form of exercise for pain reduction is aerobic, evidence has shown that submaximal isometric contractions engage a centralized pain inhibitory response such that the analgesic effect is not constrained to the exercising limb [5], [10]. Demonstrating that short-duration submaximal contractions can influence the perception of pain that is simultaneously experienced in another limb is important because this approach may translate to clinical rehabilitation settings. However, to date, most studies have examined pain perception before and after relatively long isometric contractions [4], [5], [7]. Evidence for an analgesic effect when acute painful stimuli and acute isometric contractions occur simultaneously and for a similar amount of time is limited to a single study that showed a lateralized analgesic effect at one submaximal force level [10]. While encouraging, it is not known whether pain perception is related to the strength of the isometric contraction (dose-response effect of force amplitude). This gap in the literature is important because a dose-response effect of isometric force production on pain perception would influence decision making on rehabilitation regimens in clinical settings. The goal of the current study was to determine whether there is a dose-response effect of force amplitude on pain-perception.

Previous evidence has shown that changing the parameters of a force production task influences pain perception. For instance, assessment of pain thresholds before and after elbow flexion at 25% and 80% of maximum voluntary contraction (MVC) until task failure, 25% of MVC for two minutes, and three short duration MVC trials [7] showed reductions in pain ratings for all conditions aside from the 25% 2-minute contraction. The analgesic effect was greatest following the 25% contraction to task failure, suggesting that low-intensity long-duration contractions have the greatest analgesic effect. Other evidence for a dose-response effect of force production on pain perception comes from a study that examined the nociceptive reflex response and showed that pain perception was reduced during grip force contractions at 15% of MVC and 25% of MVC as compared to contractions at 1% of MVC [8]. However, the number of electrical stimuli and the duration of each isometric contraction varied across subjects. Hence, conclusions on the dose-response effect of force amplitude on pain perception are limited because previous studies have not held the duration of force contractions constant while varying force amplitude.

To address this issue we have designed a protocol that allows us to accurately guide the amplitude of an isometric pinch grip force contraction on a trial-by-trial basis, while a thermal pain eliciting stimulus is simultaneously delivered for the same duration to the non-contracting hand. We test the hypothesis that increasing the amplitude of acute isometric force production will lead to a reduction in the perception of a pain-eliciting thermal stimulus on the non-contracting hand.

## Methods

### Ethics Statement

The University of Florida’s Institutional Review Board approved the procedures involved in this study. All individuals read and signed the informed consent prior to participation.

### Subjects

Forty-two healthy right-handed adults with normal or corrected-to-normal vision and not currently taking any pain medications participated (21 Female, 21 Male; M = 21.28 yrs, range: 18–45 yrs). Exclusion criteria were any history of neurological disease, including any history of pain syndrome. Subjects who verbally self-reported any history of these disorders were excluded during prescreening. All subjects completed the State and Trait segments of the STAI Anxiety Inventory (STAI-S, STAI-T) [11], the Beck Depression Inventory (BDI), [12] Pain Anxiety Symptoms Scale (PASS), [13] Pain Catastrophizing Scale (PCS) [14], and the Tampa Scale of Kinesiophobia (TSK) [15].

### Experimental Protocol

Subjects produced force with their right hand while thermal stimuli were delivered to the thenar eminence of their left hand at the same time. For each target-force level there were both active and passive conditions. During active conditions subjects produced force with their right hand. During passive conditions subjects did not produce force but viewed a pre-recorded force-trace on the screen. Visual information was therefore consistent across active and passive conditions. Thermal stimulation was delivered during both active and passive conditions which allowed us to calculate analgesic scores while controlling for visual information between conditions.

### Force Measurement

The force transducers used were ELFF-B4 model load cells constructed from piezoresistive strain gauges measuring force up to 100 N (Measurement Specialties, Hampton, VA). Force data was collected by Coulbourn Instruments Type B V72-25B amplifiers at an excitation voltage of 5 V. The force signal was transmitted via a 16-bit A/D converter and digitized at 125 Hz. The summed output from the force transducers was presented to the subject using a visual display on the computer screen. The force output was displayed on a 40-inch LCD screen at a resolution of 1600×1024 pixels and a refresh rate of 59 Hz. The subject sat 50 inches from the screen. Force production was guided by real-time visual feedback.

### Maximum Voluntary Contraction

During the practice session, each subject’s maximum voluntary contraction (MVC) was calculated using a force transducer (Jamar Hydraulic Pinch Gauge). Subjects were asked to sustain a contraction of maximum force for three consecutive 5-s trials. Trials were separated by a 60-s period of rest. The MVC was calculated as the average of the three peak force levels. Mean MVC was 66.47 (SD  = 20.53) Newtons.

### Pain Measurement

Thermal stimuli were delivered to the thenar eminence of the left hand using a 573 mm^2^ CHEPS thermode (PATHWAY System, Medoc Advanced Medical Systems Ltd, Ramat Yishay, Israel).

### Thermal Calibration

Consistent with previous work [16], [17], the pain threshold of each subject was determined through a calibration protocol. Subjects were first made familiar with the rating scale. The scale ranged from 0 =  “no sensation” to 10  =  “intolerable pain.” Middle points were described as “warm but not painful,” (corresponding to scores of 1–3), “moderate pain,” (corresponding to scores of 4–6) and “more intense pain” (corresponding to scores of 7–9). Subjects were not shown the numbers because we wanted the scale to remain analog. The calibration paradigm consisted of 18 trials; the duration of each trial was 15 s and was preceded by 7.5- to 15-s rest duration. The data from the first two trials were always discarded. In the remaining 16 trials, each temperature from 41 to 48°C was delivered twice in a random order. After each trial, subjects rated perceived pain intensity during the trial by moving a cursor on a visual analog scale (VAS) displayed on the screen. A baseline temperature of 32°C was maintained during the rest and the rating period. For each subject, ratings were plotted against the corresponding temperatures. To qualify for the study, the relation between an individual’s ratings and the applied temperatures had to be consistent (linear regression R^2^>0.65) [16]. Data from four subjects (3 female, 1 male) were excluded from the analysis because of inconsistent ratings during the calibration period (R^2^<0.65). Hence, 38 participants (18 female, 20 male) were included in the analyses. The mean R^2^ value was 0.85 (SD = 0.08). Warm and moderately painful temperatures were determined individually based on a VAS value of 2 (warm) and 6 (moderately painful). For each individual we took the corresponding temperature for each of these VAS values and rounded to the nearest 0.5 degrees. The mean warm-temperature was 42.51°C (SD = 1.36) and the mean hot temperature was 45.42°C (SD = 1.17). Mean scores for questionnaire measures are shown in Table 1.

**Table 1:** Mean Questionnaire data.

Questionnaire	M	SD
BDI	2.34	4.06
PASS	56.66	24.62
PCS	11.87	9.80
STAIT	29.87	11.28
TRAIT	32.18	7.38
TSK	14.74	11.61

BDI  =  Beck Depression Inventory. PASS  =  Pain Anxiety Symptoms Scale. PCS  =  Pain Catastrophizing Scale. STAIT and TRAIT  =  State and Trait segments of the STAI Anxiety Inventory TSK  =  Tampa Scale of Kinesiophobia. M  =  Mean. SD  =  Standard Deviation.

### Experimental Task

**Figure 1:**
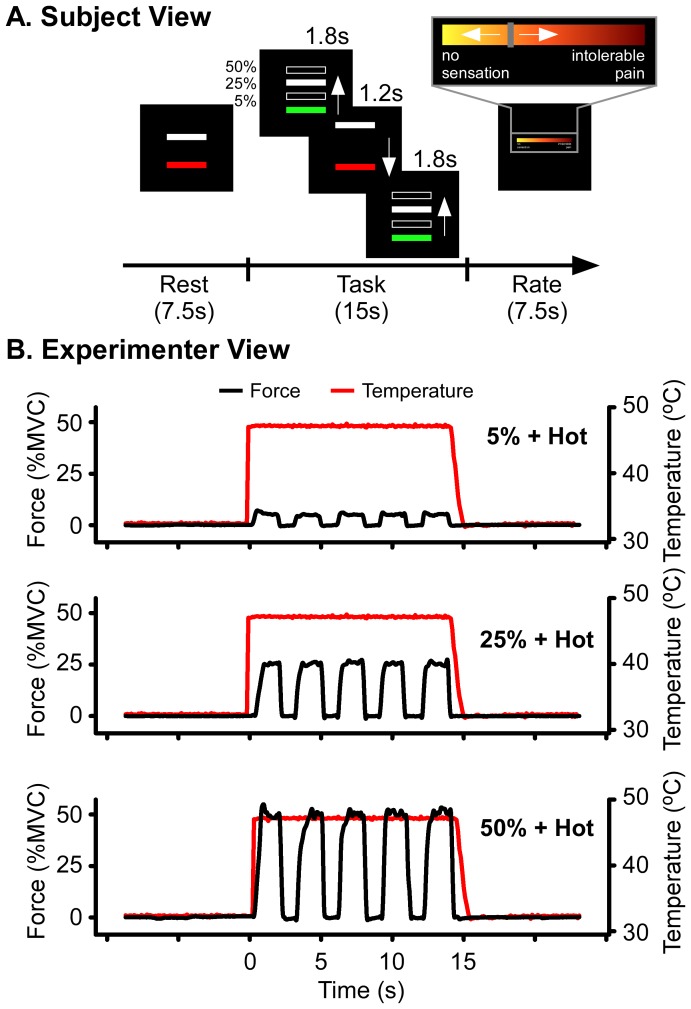
Experimental paradigm. **A.** The white bar represented the target force level which was set at the beginning of each trial to 5%, 25%, or 50% of each subjects’ MVC. A second red/green force bar was controlled by the subject and provided real-time visual feedback of their force production during active conditions, or moved according to a pre-recorded force trace during passive conditions. The bar was red during rest periods and green when the subject had to produce force or view the pre-recorded force trace. When the force bar turned green, the subject’s goal was to match the green bar with the white bar. Each trial was 15s long, and included 5 force pulses. Each pulse was 1.8s (bar turns green) and pulses were separated by a 1.2s rest period (bar turns red). Trial timing was the same during passive conditions but subjects were instructed to view the green bar and not produce any force. The pre-recorded force trace was collected during each participant’s practice session. Each 15s trial was followed by a 7.5s rating period. Subjects rated the level of stimulation they experienced during the previous 15s trial. Ratings were made using a button box to control a cursor on a visual analog scale (VAS) presented on the screen. **B**. Example force traces (black lines – left y-axis) and temperature traces (red lines – right y-axis) from active trials from one subject for the hot condition at 5%, 25%, and 50% of MVC.

Figure 1A shows the experimental paradigm. Consistent with previous work, we used a visually guided grip force task [18], [19], [20], [21], [22]. Two bars were always visible to the subject. The white bar represented the target force level and at the beginning of each trial the bar was set at 5%, 25%, or 50% of each subjects’ MVC. A second red/green force bar was controlled by the subject and provided real-time visual feedback of their force production during active conditions, or moved according to a pre-recorded force-trace during passive conditions. The bar was red during rest periods and green when the subject had to produce force or view the pre-recorded force trace. When the force bar turned green, the subject’s goal was to match the green bar with the white bar. Each trial was 15 s long, and included 5 force pulses. Each pulse was 1.8 s long (bar turns green) and pulses were separated by a 1.2-s rest period (bar turns red). Trial timing was the same during passive conditions but subjects were instructed to view the green bar and not produce any force. The pre-recorded force trace was collected during each participant’s practice session. During the practice session subjects produced force at each target level while experiencing warm and moderately painful temperatures. Pre-recorded force trials were played back to the subject during the passive condition for the corresponding active condition. This meant that the passive condition could occur first in the experimental paradigm and order effects were avoided. Because of the relative ease of the task, subjects were able to perform the task accurately during the practice session after two trials. Each 15-s trial was followed by a 7.5-s rating period. Subjects rated the level of stimulation they experienced during the previous 15-s trial. Ratings were made using a button box to control a cursor on a VAS presented on the screen which is also shown in Figure 1A. The range of the rating bar was 0-10 with a resolution of 0.1. During experimental trials, 2 verbal descriptors were visible to the subject: “no sensation” on the left side of the scale and “intolerable pain” on the right side of the scale. Mean ratings for the baseline, warm, and hot temperatures were 0.35 (range: 0 – 3.63), 1.76 (range: 0 – 5.67), and 6.78 (range: 1.6 – 9.27) respectively. Figure 1B shows example force traces (black lines – left y-axis) and temperature traces (red lines – right y-axis) for the hot condition at 5%, 25%, and 50% of MVC.

Subjects completed a total of 6 blocks (3 active, 3 passive) of experimental trials. Two independent variables were manipulated within each block: 1) temperature of thermal stimulation, and 2) target force. Thermal stimulation during each trial was either at a baseline temperature (3 trials), a warm temperature (3 trials), or a pain-eliciting temperature (3 trials) and target MVC was either at 5% (3 trials), 25% (3 trials), or 50% (3 trials). Within each block, every combination of temperature and target force was performed once, totaling 9 trials. The order of the trials within a block was pseudorandomized with the constraint that the painful temperature was never presented twice in a row. The sequence used in each active block was repeated in a corresponding control (passive) block. The order of the blocks was pseudorandomized across subjects with two constraints - active and passive blocks were always interleaved, and an active block and its control (passive) block always appeared in succession (passive block appeared first in half the subjects). A 7.5-s rating period followed each 15-s trial, and a 7.5 to 15-s rest period followed the rating period. Consistent with previous evidence [10], a 60-s rest interval separated trials 4 and 5 to provide a standardized rest period within each block and to help avoid habituation, sensitization, and fatigue affects.

### Statistical Analysis

**Rating data.** Thermal stimulation ratings were made after every trial. Mean rating scores were calculated from all trials in each condition. To control for potential differences in absolute ratings between force amplitudes, analgesic scores were calculated by subtracting ratings during each active condition from ratings during its corresponding control (passive) condition. To test our experimental hypothesis we examined differences in analgesic scores in a 2-way ANOVA (amplitude: [5%, 25%, 50%] × temperature [baseline, warm, hot]). Significant interactions and main effects were followed up with simple effects tests and Bonferroni corrected paired t-tests to compare analgesic scores between force amplitudes separately for each temperature.

**Grip force data.** Force data was analyzed using custom algorithms in LabVIEW. The force-time series data were digitally filtered with a fourth-order Butterworth filter with a 20 Hz low-pass cut-off. Force production was characterized by measures of mean force amplitude, force variability (standard deviation), and relative force variability (coefficient of variation). Force amplitude and force variability were extracted from the middle 1 s of each pulse. The middle 1 s of the pulse was calculated following identification of the onset and offset of each pulse. The onset of each contraction was identified as the time point where force rose above two times the baseline value. The offset of each contraction was identified as the next time point where force fell below two times the baseline value. Baseline values were calculated as the mean force amplitude during the 300 ms prior to each 15-s trial. The summary statistic for each force-dependent variable was the mean score calculated from all the pulses in each condition. Separate two-way (temperature [baseline, warm, hot] × target force level [5%, 25%, and 50% of MVC]) repeated measures ANOVAs were run for each dependent variable.

For analyses in which the sphericity assumption was violated, Greenhouse–Geisser degrees of freedom corrections were applied and are reported. Significance level was set at p<0.05.

## Results

### Rating Data

**Figure 2:**
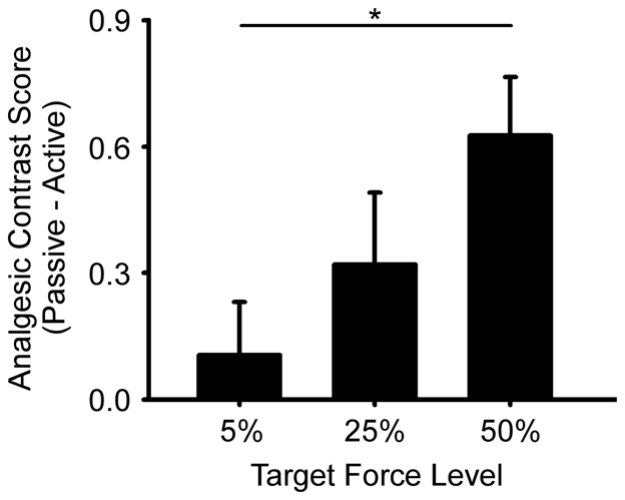
Analgesic score (passive condition – active condition) are shown for each target force level for the hot condition. Analgesic scores at 50% were greater than at 5% of MVC. Error bars represent SE.

The primary aim of the current study was to examine the dose-response effect of target force amplitude on the perception of a pain-eliciting stimulus. Table 2 shows the mean and standard deviation of rating data for all temperatures for the passive and active trials at the 5%, 25%, and 50% target-force levels. Analgesia scores were calculated and compared across all target force levels and temperatures. A main effect of temperature (F (1.68, 59.48)  = 7.14, p = 0.003) was superseded by a target force level × temperature interaction (F (2.97, 109.72)  = 2.78, p = 0.045). The significant interaction was followed up with simple effects tests at each temperature. At the hot temperature, pain ratings differed as a function of target force level (F (2, 74)  = 5.14, p = 0.008). Figure 2 shows the contrast values for each target force level at the hot temperature. Paired t-tests found that the analgesic effect at 50% of MVC was greater than at 5% of MVC (t (37)  = –3.180, *p* = 0.003). Although there was a progressive increase in the analgesic effect with an increase in force amplitude, ratings at 25% were not significantly different from ratings at 50% of MVC (t (37)  = –1.696, *p* = 0.098) or 5% of MVC (t (37)  = –1.495, *p* = 0.143). We also compared each analgesic score to zero. The analgesic score was significantly greater than zero at 50% (t (37)  = 4.53, *p*<0.001), but not at 25% (t (35)  = 1.864, (p = 0.070) or 5% (t (37)  = 0.828, *p* = 0.413). Simple effects tests at the baseline temperature and the warm temperature were not significant (all p’s>0.05).

**Table 2:** Mean rating data for passive trials and active force trials for each temperature and for each target force level.

		Target Force Level					
		5%		25%		50%	
		M	SD	M	SD	M	SD
Passive Trials							
	Base	0.32	0.63	0.41	0.74	0.27	0.47
	Warm	1.58	0.93	2.15	1.16	1.98	1.19
	Hot	6.93	1.68	6.74	1.64	7.19	1.34
Active Trials							
	Base	0.28	0.51	0.44	0.73	0.39	0.81
	Warm	1.40	0.91	1.87	1.17	1.58	1.22
	Hot	6.83	1.62	6.42	1.53	6.57	1.56

M  =  Mean. SD  =  Standard Deviation.

### Grip Force Task

**Figure 3:**
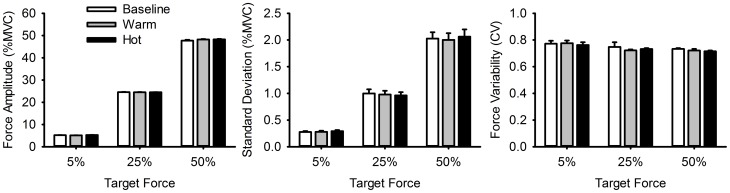
Mean amplitude and variability of force production during active trials. Figures A-C show mean force amplitude, mean standard deviation, and mean coefficient of variation of force production during active trials for each temperature and each target MVC level. Error bars represent SE.

Figure 3 and Table 3 show the amplitude of force production, the standard deviation of force production, and the coefficient of variation of force production for each temperature and each target force level. The amplitude of force production differed as a function of the target force (F (1.17, 42.21)  = 51765.91, p<0.001). An increase in target force level corresponded with an increase in the amplitude of force production, and force amplitude significantly differed between all target force levels (all p’s<0.001). The amplitude of force production did not differ as a function of temperature (*F* (1.51, 54.32)  = 1.42, *p*>0.05), and there was no interaction between target force level and temperature (*F* (1.71, 61.36)  = 2.24, *p*>0.05). The standard deviation of force production differed as a function of target force level (F (1.24, 44.45)  = 262.43, p<0.001). Follow-up tests found that standard deviation scores for each target force level increased parametrically across the target force levels and were all significantly different from each other (all p’s<0.001). No main effect of temperature was found (F (2, 74)  = 0.148, p>0.05), and there was no interaction between target force level and temperature on the standard deviation of force production (F (2.27, 81.69)  = 0.29, p>0.05). Coefficient of variation (CV) varied as a function of force level (F (1.29, 47.72)  = 4.55, p<0.05), with higher CV scores found for the 5% target as compared to the 50% target (p = 0.01). CV did not differ as a function of temperature (F (1.26, 46.46)  = 1.26, p>0.05), and there was no interaction between target force level and temperature (F (1.51, 55.83)  = 0.26, p>0.05).

**Table 3:** Mean force amplitude, standard deviation, and coefficient of variation of force production at each temperature and each target force level.

		Target Force Level					
		5%		25%		50%	
		M	SD	M	SD	M	SD
Force Amplitude							
	Base	5.22	0.34	24.55	0.65	47.72	2.02
	Warm	5.12	0.30	24.48	0.58	48.00	1.87
	Hot	5.29	0.39	24.48	0.47	48.23	1.27
Standard Deviation							
	Base	0.28	0.10	1.00	0.47	2.03	0.71
	Warm	0.28	0.13	0.99	0.42	2.00	0.76
	Hot	0.31	0.15	0.96	0.36	2.06	0.81
Coefficient of Variation							
	Base	0.77	0.13	0.75	0.21	0.73	0.04
	Warm	0.78	0.13	0.72	0.04	0.72	0.07
	Hot	0.76	0.13	0.73	0.04	0.72	0.04

## Discussion

The goal of the current study was to examine the dose-response effect of force amplitude of an isometric contraction on pain perception. A pinch-grip force task was completed with the right hand to different target force amplitudes while pain-eliciting thermal stimulation was simultaneously delivered to the thenar eminence of the left hand. Consistent with our hypothesis, our results show that an increase in the amplitude of force produced by one hand corresponded with a decrease in pain perception in the other hand. Our observations provide novel evidence that the centralized pain inhibitory response that underlies analgesia is sensitive to and enhanced by strong isometric contractions.

Contractions that are minutes long have previously been shown to lead to a reduction in pain perception in one limb during or after isometric contractions of the opposite hand [5], [6]. We recently extended these findings by showing that much shorter 15-s contractions also lead to a reduction in perception of a painful thermal stimulus that is simultaneously delivered to the other non-contracting hand [10]. Together these findings suggest that a centralized pain inhibitory response underlies the analgesic effects associated with isometric contractions, but the dose-response characteristics of this association are not clear. Holding the amplitude of a contraction constant at 25% of MVC and varying its duration between 1, 3, and 5 minutes does not alter the magnitude of the analgesic effect, although each condition is effective in reducing the perception of pain [23]. However, other observations show that the amplitude and duration of the contraction do alter pain perception, with 25% of MVC contractions to task failure leading to significant analgesic effects, whereas contractions at 25% of MVC for 2 minutes do not. This finding suggests that the recruitment of high threshold motor units are necessary for the analgesic effect to emerge, and that increasing force amplitude in acute tasks is not effective in reducing pain perception [7]. However, other evidence suggests that the recruitment of high threshold motor units is not necessary for analgesic effects to emerge. For instance, reductions in pain perception have been demonstrated during and following shorter contractions in the range of 15 to 120 s [5], [6], [10]. Other findings suggest that ∼4.5 minute long grip force contractions to 15% and 25% of MVC lead to a reduction in pain perception relative to contractions at 1% of MVC, and that this reduction in pain perception is related to increased blood pressure; consistent with an arterial baroreceptor inhibition mechanism [8]. However, changes in the length of contractions between conditions, the inclusion of ratings to non-pain eliciting stimuli, as well as other evidence showing that blood pressure may not relate to analgesia [23], demonstrate that mechanisms underlying the relationship between force production and pain perception are not straight forward.

Observations from animal and human work suggest that release of endogenous opioids and activation of a central nociceptive descending inhibitory system are two possible mechanisms that may account for why force production alters pain perception. Muscle contractions activate primary afferents, and stimulation of afferent fibers in skeletal muscle can result in activation of endogenous opioid and non-opioid pain inhibitory mechanisms which may scale in activity with an increase in contraction strength. Motoneurons are recruited in order of increasing size [24], and increases in the amplitude of isometric force contractions are associated with an increase in the number and firing rate of motor units recruited [25]. In addition to changes in the motor neuron pool at the spinal level, increasing the amplitude of a force contraction is associated with region specific increases in brain activity. Specifically, functional magnetic resonance imaging evidence has shown that increases in the amplitude of an isometric grip force contraction correspond with an increase in the blood-oxygen-level-dependent (BOLD) signal in the primary motor cortex, ventral thalamus, basal ganglia, and cerebellum [26], [27]. Importantly the BOLD signal in primary motor cortex was not different between 5% and 20% of MVC conditions, with target force having to increase to 40% for differences in the BOLD signal to scale with a change in force amplitude. While this finding provides insight into the sensitivity of the BOLD signal, it also suggests that quite large differences in the amplitude of force are necessary to detect significant changes in pain perception. This interpretation dovetails nicely with the current findings and previous evidence to suggest that relatively large differences in force amplitude are necessary to detect a significant change in pain perception [8].

We analyzed the amplitude of force production to examine how well each participant produced the required target force in each condition. We also examined the standard deviation and coefficient of variation of force production across target levels (5%, 25% and 50%). Although both the mean amplitude and the standard deviation of force output increased with an increase in target force level [28], [29], the coefficient of variation decreased, revealing a relative increase in variability at low relative to high target forces, which is consistent with previous evidence [30].

Electrical or magnetic stimulation of primary motor cortex has been associated with analgesia, leading to the suggestion that the primary motor cortex is an entry port for the modulation of pain processing circuits [31]. Animal work has shown that activation of the motor cortex and corticospinal tract inhibits spinothalamic neurons in monkey [32] and inhibits the response of spinal dorsal horn neurons to noxious, but not innocuous mechanical stimulation in rat [33]. Although future studies are necessary to determine whether changes in motor unit recruitment, firing rate, and increased activity in brain and spinal cord directly relate to pain perception in humans, our observations do show that increasing the amplitude of an acute voluntary grip force contraction leads to a decrease in pain perception. These observations are important because they show that analgesic effects during acute 15 s contractions are sensitive to force amplitude, and that this effect cannot be accounted for by differences in the duration of the contraction across target amplitudes, differences between the duration of the contraction and pain-eliciting stimulus, and differences in visual information across experimental conditions.

The current study has a number of limitations. First, the same scale was used to measure both nociceptive and non-nociceptive stimulation. Second, the magnitude of the analgesic effect in the current study may be considered modest. While the effect size we show is similar to that shown using working memory [16] and motor [10] tasks, it is possible that the motor analgesic effect may be further enhanced by increasing the duration of the contraction or altering the pattern of the contraction. Increasing the duration of the contraction, however, would likely lead to fatigue which may engage a different mechanism from the one underlying the current findings.

In summary, our findings advance our current understanding of the relation between voluntary motor system engagement and pain perception in humans. Our experimental paradigm and findings are consistent with cognitive analgesia studies which have shown that when a pain eliciting stimulus is delivered during an acute working memory task, pain perception scales with a change in the memory load [17], [34]. That is, a more difficult working memory task is associated with a greater reduction in pain perception [34]. It is important to note that isometric contractions at 50% as compared to 5% of MVC are not more difficult, but instead elicit functional changes in the muscle, brain, and spinal cord. We show that these changes influence pain perception in a dose-dependent manner and therefore have implications for rehabilitation because the amplitude of force produced by one hand can reduce the perception of pain in the non-contracting hand. Future studies are necessary to determine whether the dose-response effect of force amplitude on analgesia extends to individuals with endogenous pain, but the current findings provide a foundation and paradigm upon which to build such studies.
